# Stem Cell–Related Gene CALR as a Novel Prognostic Factor for Bladder Cancer: Implications for Immunotherapy

**DOI:** 10.1155/humu/5569005

**Published:** 2025-12-17

**Authors:** Zhihao Ling, Shuo He, Tianyu Li, Jiandong Zhang, Beibei Liu

**Affiliations:** ^1^ Department of Urology, The First Affiliated Hospital of Bengbu Medical University, Bengbu, China, bbmc.edu.cn

**Keywords:** BLCA, CALR, CSC, machine learning, prognosis

## Abstract

Cancer stem cells (CSCs) are a unique category of cells located within tumors, characterized by their exceptional self‐renewal abilities and capacity to differentiate into different types of tumor cells. These cells are crucial in processes such as tumor metastasis, recurrence, and resistance to treatment. Nevertheless, their particular roles in bladder urothelial carcinoma (BLCA) require deeper exploration. This investigation firstly assessed the relationships between genes associated with CSCs and both the prognostic outcomes and responses to immunotherapy in patients with BLCA using cluster analysis methods. Among the genes linked to stem cells, CALR was identified as the most notable prognostic marker by the XGBoost algorithm. Additionally, the study explored the correlation between CALR and immune cell infiltration, as well as its interaction with mitomycin using molecular docking methods. In vitro experiments further validated that CALR affects BLCA stem cells. Utilizing multiple machine learning approaches, this study identified 14 essential stem cell–associated genes, underscoring their significance for BLCA diagnosis and potential therapeutic targeting. Critically, CALR demonstrates a strong correlation with prognostic outcomes and immunotherapy responses in BLCA, consistent with experimental findings indicating its elevated expression in BLCA and association with poor prognosis. Laboratory investigations have demonstrated that reducing CALR expression can lessen the stemness features of BLCA. Our results highlight the critical role of genes related to stem cells in BLCA and identify CALR as a promising target associated with stem cell functionality.

## 1. Introduction

Bladder urothelial carcinoma (BLCA) is a prevalent cancer affecting men worldwide and represents a significant public health challenge due to its high incidence and mortality rates. Treatment options encompass surgery, radiation therapy, immunotherapy, and other modalities [1]. However, patient outcomes for this malignancy remain poor, largely due to challenges such as disease recurrence, metastatic spread, and resistance to drug therapy [2]. Contemporary management strategies demonstrate optimal efficacy primarily in patients identified during early disease stages; conversely, therapeutic options are significantly limited for those presenting with late‐stage diagnoses. Given the pivotal role of genomic alterations in BLCA development and progression, a comprehensive investigation into the disease′s underlying biological mechanisms is essential [3]. This research is crucial for discovering novel molecular targets for therapeutic intervention. BLCA is significantly influenced by cancer stem cells (CSCs), which play a crucial role in its progression, development, and resistance to treatment. Studies reveal that BLCA is a particularly complex and heterogeneous stem cell pathology. Untreated cases can result in elevated incidence and mortality rates [4]. CSCs possess the unique abilities to self‐renew, metastasize, and evade conventional chemotherapy, positioning them as key contributors to tumor growth [5]. The diversity of CSCs within BLCA presents a considerable obstacle, underscoring the necessity for more effective therapies aimed at these specific stem cell groups. Not only are CSCs in BLCA implicated in tumor development and escalation, but they are also strongly associated with resistance to chemotherapy. Research suggests that BLCA CSCs retain their stem cell–like traits by activating the Wnt/*β*‐catenin signaling pathway, which contributes to their drug resistance capabilities [6]. Additionally, the existence of CSCs in BLCA correlates with increased cisplatin resistance and tumor advancement, as these cells may promote malignancy by upregulating Bmi1 and Nanog expressions [7]. The presence of CSCs significantly constrains the efficiency of standard treatments for BLCA. Evidence indicates that CSCs may conserve their stem cell–like properties via the IL6/IL6R/STAT3 signaling pathway, suggesting that targeting this pathway could be a therapeutic strategy against CSCs [8]. Furthermore, research has uncovered that BLCA CSCs could facilitate epithelial–mesenchymal transition (EMT) through the SHH signaling pathway, thus boosting both the invasiveness of the tumor and its resistance to drugs [9]. To gain a deeper insight into the properties of CSCs associated with BLCA, scientists have investigated their origins and evolutionary processes. It is suggested that CSCs in BLCA may derive from standard bladder stem cells, which experience malignant changes due to the influence of the tumor microenvironment [10]. Moreover, these CSCs are thought to have the ability to manage their self‐renewal and tumorigenic potentials via certain signaling pathways, including the KMT1A‐GATA3‐STAT3 circuit [11]. In conclusion, CSCs are essential in the development, progression, and resistance to treatment in cases of BLCA. Investigating CSCs not only deepens our comprehension of the biological processes that drive BLCA but also establishes crucial theoretical groundwork for the formulation of innovative therapeutic approaches. Targeting CSCs alongside their related signaling pathways may lead to the development of more efficient treatment alternatives for individuals diagnosed with BLCA.

The CALR encodes calreticulin, a multifunctional endoplasmic reticulum (ER) protein. Calreticulin is essential as a molecular chaperone for glycoprotein folding and ER quality control. It also plays critical roles in immune modulation, phagocytosis, and cell signaling [11]. Using machine learning, our study conducted a comprehensive analysis of stem cell–related genes in BLCA and identified the functionally significant gene CALR.

## 2. Materials and Methods

### 2.1. Datasets and Patient Samples

This investigation utilized transcriptomic datasets from bladder carcinoma patients sourced from The Cancer Genome Atlas (TCGA) and the Gene Expression Omnibus (GEO) repositories. Specifically, the TCGA‐BLCA (https://portal.gdc.cancer.gov/) cohort comprised 406 tumor specimens and 19 samples of histologically normal bladder tissue. Additionally, the GSE13507 dataset (https://www.ncbi.nlm.nih.gov/geo/query/acc.cgi?acc=GSE13507), obtained from GEO, included 165 primary urothelial carcinoma cases and nine nonmalignant bladder controls [12].

### 2.2. Negative Matrix Factorization (NMF) Cluster Analysis

The NMF technique is used to uncover biologically meaningful coefficients in the gene expression matrix, effectively arranging genes and samples to emphasize the fundamental structural features of the data, thereby aiding in sample classification [13]. A comparative examination of differential expression between Clusters A and B was carried out using the “limma” R package, following the parameters of |logFC| > 0.5 and an adjusted *p* value of < 0.05. Afterward, the “NMF” R package was applied to categorize all samples based on the differentially expressed genes (DEGs) identified in the subclusters, aiming to reveal potential molecular subtypes. The “brunet” algorithm was utilized, performing 100 iterations for each designated value within the cluster range of 2–10. The ideal number of clusters was established by assessing cophenetic correlation, dispersion, and silhouette width [14, 15].

### 2.3. Immune Infiltration

To achieve a thorough evaluation of immune infiltration scores, we utilized the R package known as immunedeconv [16]. We meticulously reviewed all algorithms offered by this tool, each showcasing distinct analytical advantages. For the current study, we opted for the CIBERSORT methodology because it can assess a broader spectrum of immune cell types [17].

### 2.4. Cell Culture

The human bladder cancer cell lines 647‐V (RRID: CVCL_1049) and J82 (RRID: CVCL_0359) were acquired in 2022 from the Cell Bank of the Committee on Type Culture Collection of the Chinese Academy of Sciences, located in Shanghai, China. Both 647‐V and J82 cells were cultured in 1640 medium, supplemented with 10% fetal bovine serum. The cells were incubated in a humidified environment at 37°C with 5% carbon dioxide. We confirmed that the cell lines used were free from contamination and were accurately identified.

### 2.5. Transfection of siRNAs

The small interference fragments in this study were synthesized by Shanghai Jima Company/China, CALR‐siRNA, and the specific sequences are shown in Table 1.

**Table 1:** Interference fragment sequences.

**Fragment name**	**Sense sequence (5′-3′)**	
CALR‐siRNA1	GUGACGAGGAGAAAGAUAATT	UUAUCUUUCUCCUCGUCACTT
CALR‐siRNA2	GCAAGAACGUGCUGAUCAATT	UUGAUCAGCACGUUCUUGCTT
CALR‐siRNA3	GUGAAGCUGUUUCCUAAUATT	UAUUAGGAAACAGCUUCACTT

### 2.6. Quantitative Real‐Time PCR Analysis

The running program settings were selected according to the experimental instrument (LightCycler 480 system), and the conditions were as follows: predenaturation: 95°C predenaturation for 30 s (1 cycle); PCR: analysis mode was quantitative analysis, denaturation at 95°C for 5 s, annealing at 60°C for 30 s (40 cycles), extension at 72°C for 30 s, 40 cycles; dissolution: analysis mode was dissolution curve, 95°C for 5 s, 60°C for 1 min, 95°C for 1 cycle; and cooling: 50°C for 30 s, 1 cycle. The primers in quantitative real‐time PCR were as follows: CALR, 5′‐CGCTTTTATGCTCTGTCGGC‐3′ (forward) and 5′‐GTGCATGTCTGTCTGGTCCA‐3′ (reverse).

### 2.7. Wound Healing Assay

According to the cell transfection and cell steps, prepare the 6‐well plate of experimental cells required for the scratch experiment, place it under a microscope to observe the cell state, and wait until the cell density grows to about 80%–90% before conducting subsequent experiments. Prepare the experimental items and 10 *μ*L pipette tips in the clean bench in advance. Place the 6‐well plate cover vertically on the 6‐well plate after ignition, and then use a 10 *μ*L pipette tip to draw vertical and horizontal lines along the plate cover, and try to keep the speed and weight of the line unchanged to avoid the distortion of the line and uneven thickness affecting the beauty of the picture. After the waste liquid containing cell fragments is sucked by the pipette, PBS is added to the well, and the plate body is gently shaken to wash the cells two to three times to remove the scratched cell fragments; 2 mL of serum‐free culture medium is added to continue to be returned to the incubator for cultivation, and according to the experimental design time, observe and take pictures at 0, 12, and 24 h to record the experimental data of the scratch picture. Note that PBS can be used to wash once before taking each picture to remove the detached cell fragments to ensure that the background of the picture is clean and beautiful.

### 2.8. Transwell Assay

For the cell migration assay, 647‐V/J82 cells were plated in 6‐well plates at a density of 5 × 10^5^ cells/cm^2^. The conditioned medium, which contained 1% FBS, was maintained for 48 h. A sterile pipette tip measuring 200 *μ*L was utilized in conjunction with a scratch template. Consistency was maintained in both the speed and force of the scratching process. The washing procedure involved using prewarmed DPBS, which was applied three times to eliminate any free‐floating cell debris, followed by serum‐free medium for ongoing culture. Scratch images were taken and observed at 0, 24, and 48 h to gather experimental data. The experiment was repeated three times, and the ImageJ software was employed to quantify the area of the scratch and perform statistical analysis.

For the cell invasion assay, the 8 *μ*m chamber was initially coated with matrix gel and incubated at 37°C for 1 h. A suspension of 647‐V and J82 cells was then added to the chamber, while 100 *μ*L of serum‐free medium was introduced into the upper chamber to prevent the formation of bubbles that could interfere with the results. Depending on the specific experimental conditions, the appropriate conditioned medium was added to the lower chamber. After thoroughly mixing, the laboratory bench was allowed to sit for 10 min before carefully transferring the 24‐well plate to the incubator. Following a 24‐h incubation period, the chamber was removed, and the medium in the upper section was discarded. The upper chamber was gently wiped with a cotton swab to eliminate any remaining cells. The chamber was then washed twice with PBS, fixed with 600 *μ*L of 4% paraformaldehyde per well at room temperature for 30 min, and subsequently washed two more times with PBS. Crystal violet staining was performed for 30 min at room temperature. After two to four additional washes with PBS, the chamber was inverted to dry, and images were captured for cell counting. This process was repeated three times, with ImageJ utilized to quantify the cell count and perform statistical analysis [15].

### 2.9. Immunofluorescence Assay

Cells were placed onto sterile slides and subsequently transferred into 24‐well plates. Following the addition of complete medium, the cells were cultured until reaching a density of approximately 70%–80%. A triple treatment for fixation, permeabilization, and blocking was conducted: The medium was removed, and 1 mL of precooled 4% paraformaldehyde was introduced per well, followed by fixation at 4°C for 30 min. The cells were then washed three times with PBST for 5 min each. A permeabilization solution of 0.1% Triton X‐100 (containing 0.1% sodium citrate) was applied for 30 min at room temperature, followed by three additional washes with PBST for 5 min each. Blockage was performed using 1% BSA for 1 h, after which specific primary antibodies were introduced and incubated in a wet box at 4°C for 12–16 h. The primary antibodies were subsequently retrieved and washed three times with PBST (5 min each). An Alexa Fluor 550‐labeled secondary antibody (diluted 1:500) was added next and incubated in the dark for 60 min. The samples were counterstained with DAPI (1 *μ*g/mL) for 5 min and finally washed three times with PBST for 5 min. The “hanging drop method” was used to add 8 *μ*L of antifade agent to avoid bubble formation and to seal the slides. The Zeiss LSM 900 confocal system was used to collect images. Key observations, including cytoskeleton morphology (DAPI nuclear localization), SOX2‐specific fluorescence signal intensity and distribution, the morphology of cells, the expression changes of SOX2, and POU5F1‐specific fluorescence signal intensity and distribution, were observed under a fluorescence microscope.

### 2.10. Sphere Formation Assay

After digestion, the 647‐V/J82 cells in good condition were collected by centrifugation, washed twice with PBS, and cultured with ultralow adsorption culture plates (1 × 10^3^ per well) after cell counting. After 10 days, the cell spheres were observed. Then, the cell spheres were collected by filtration with a 70 *μ*m cell sieve and cultured with ultralow adsorption cell culture plates for the second round of sphere formation (second‐generation spheres). Photos were taken, and the number of second‐generation spheres was counted under a microscope.

### 2.11. Statistical Analysis

The levels of expression for genes associated with stem cells in both BLCA and normal tissues were assessed through the Wilcoxon rank‐sum test. Additionally, a prognostic analysis was performed using the log‐rank test. A *p* value below 0.05 was determined to be the cutoff for statistical significance.

## 3. Result

### 3.1. Identification of BLCA Stem Cell–Related Genes

Utilizing the TCGA‐BLCA dataset, we performed a differential analysis by dividing samples into tumor and adjacent normal tissues, which led to the identification of 944 genes with increased expression and 1450 genes with decreased expression (Figure 1a,b). We then queried the GeneCards database for genes associated with stem cells, employing “stem cell” as our search term and filtering for genes that scored above 10, yielding a total of 7186 genes. Following this, we pinpointed 14 stem cell–related genes exhibiting upregulation in BLCA that may function as prognostic indicators (Figure 1c). The expression variances among these 14 stem cell–related genes in BLCA were depicted through box plots (Figure 1d,e). Furthermore, we presented prognostic forest plots for overall survival (OS) and progression‐free survival (PFS) associated with these 14 genes in BLCA (Figure 1f,g). Based on the aforementioned results, we identified a total of 14 prognostic stem cell markers linked to the prognosis of BLCA.

Figure 1A total of 14 genes associated with stem cells have been recognized as significant prognostic indicators for BLCA. (a) Volcano plot illustrating differentially expressed genes. (b) Heatmap representation of genes with differential expression. (c) Venn diagram illustrating stem cell–related genes that have differential prognostic significance. (d, e) Analysis of expression levels of stem cell–related genes. (f) Relationship between stem cell–related genes and overall survival in patients with BLCA. (g) The relationship between stem cell–related genes and progression‐free survival in individuals diagnosed with BLCA.  ^∗∗∗^ *p* < 0.001.

**Figure:**
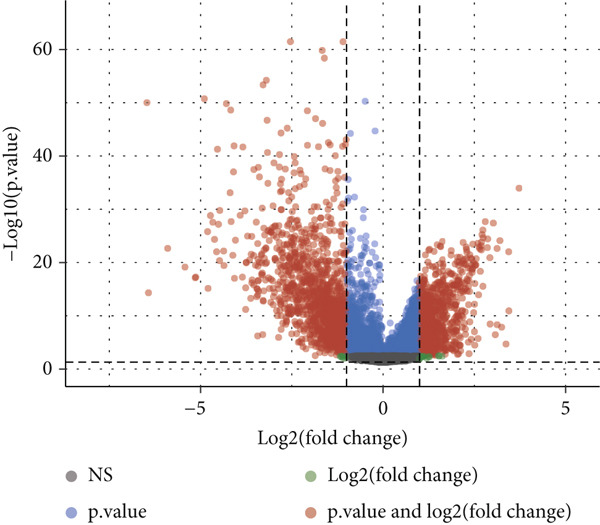
(a)

**Figure:**
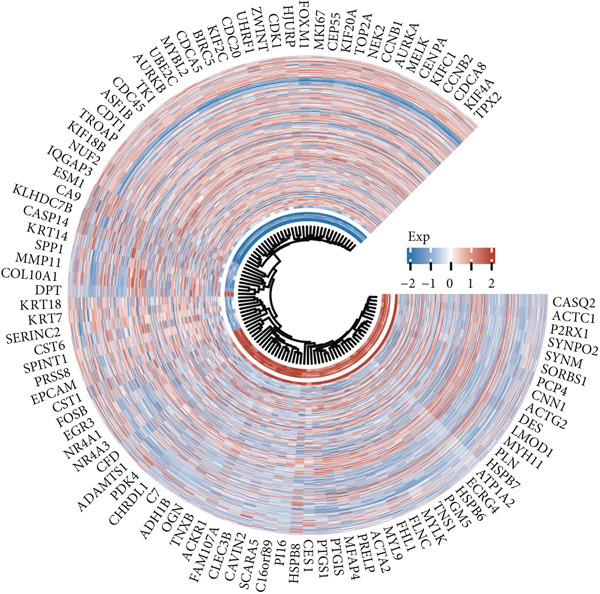
(b)

**Figure:**
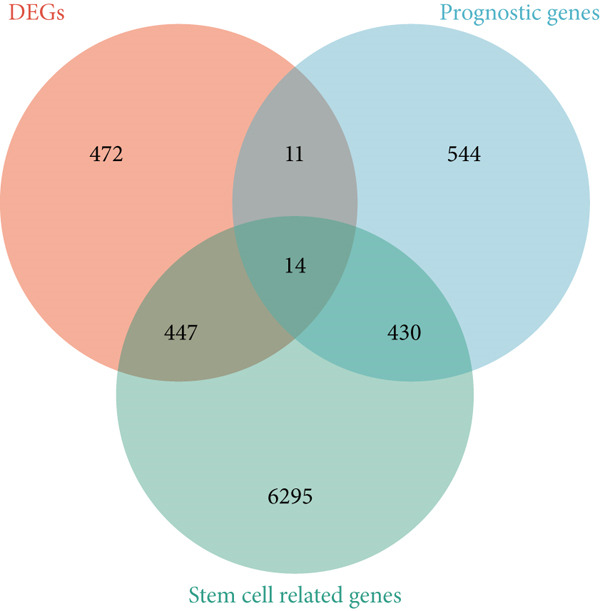
(c)

**Figure:**
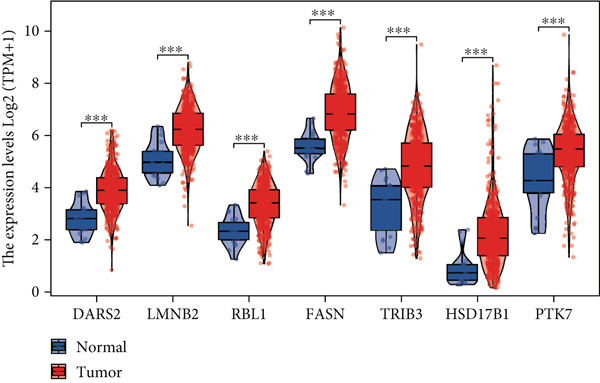
(d)

**Figure:**
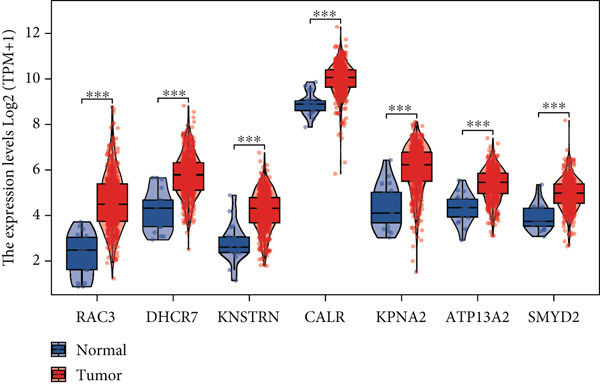
(e)

**Figure:**
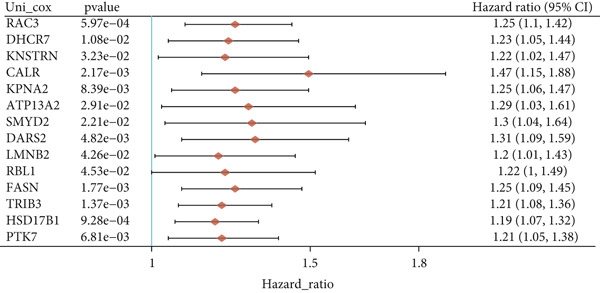
(f)

**Figure:**
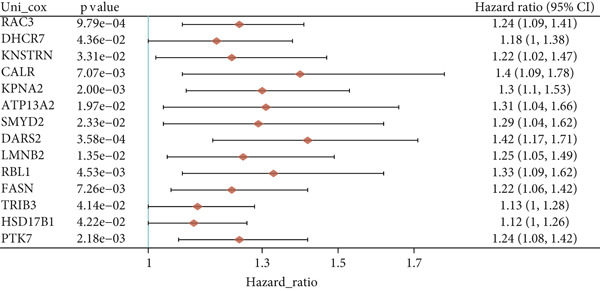
(g)

### 3.2. Functional Analysis of Stem Cell–Related Genes

The mutational profiles of genes within TCGA‐BLCA were first investigated. An oncoplot facilitated the visualization of the SNV status for the 10 most frequently mutated genes out of a total of 14 genes evaluated in BLCA. Notably, the genes ATP13A2 and FASN displayed the highest frequencies of mutation (Figure 2a). A summary of the SNV categories within the gene sets revealed a significant prevalence of missense mutations in BLCA (Figure 2b). To illustrate mutation frequencies for these genes, a heatmap was generated, pinpointing ATP13A2, RBL1, and FASN as the Top 3 with the highest mutation incidences (Figure 2c). In addition, the analysis encompassed the profiles of both heterozygous and homozygous CNVs among the 14 genes in BLCA, with circle size positively correlated with frequency (Figure 2d,e). The research further delved into methylation differences between BLCA samples and normal tissues, alongside examining the relationship between methylation patterns and mRNA expression of these genes. In the visual representation, larger circles indicated more robust correlations with methylation levels (Figure 2f,g). Furthermore, the investigation extended to the connection between these genes and various chemotherapy agents, utilizing the GDSC database, which uncovered a strong correlation involving RBL1 and the examined chemotherapy drugs (Figure 2h). Ultimately, this assessment investigated the relationships between the levels of expression of these genes and recognized biological pathways, highlighting the important link between genes related to stem cells and the stimulation of cell cycle mechanisms (Figure 2i). In conclusion, this thorough analysis clarified the possible functions of these genes in BLCA from multiple viewpoints.

Figure 2Stem cell–related genes play a vital role in BLCA. (a) Oncoplot displays the status of single‐nucleotide variants (SNVs) in the 10 most frequently mutated genes. (b) The figure outlines the various classes of SNVs found in the provided gene set. (c) The figure illustrates the SNV profile. (d) The figure presents the heterozygous copy number variation (CNV) profile. (e) The figure details the homozygous CNV profile. (f) The figure highlights the differences in methylation between tumor samples and normal samples. (g) The relationship between methylation and mRNA expression is presented in the figure. (h) The figure describes the association between gene expression levels and sensitivity to GDSC drugs. (i) The figure shows the share of cancers in which the mRNA expression of particular genes may affect pathway activity.

**Figure:**
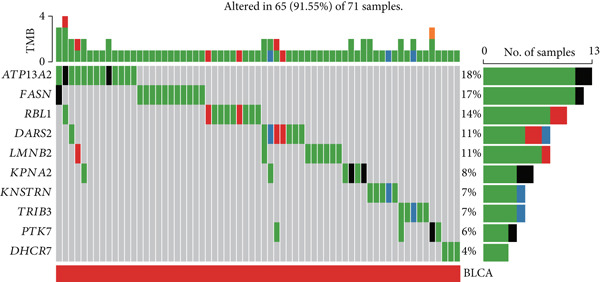
(a)

**Figure:**
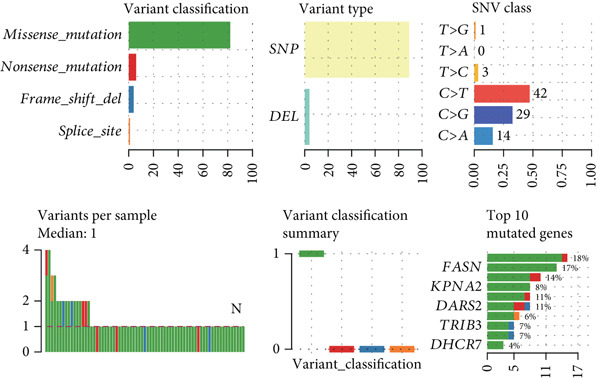
(b)

**Figure:**
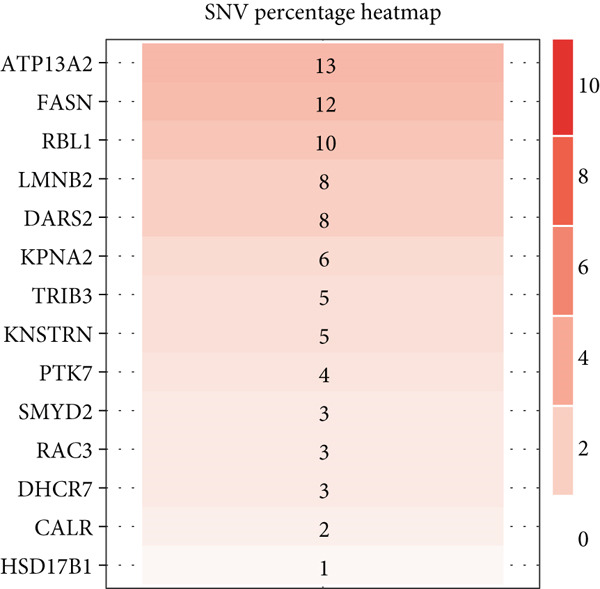
(c)

**Figure:**
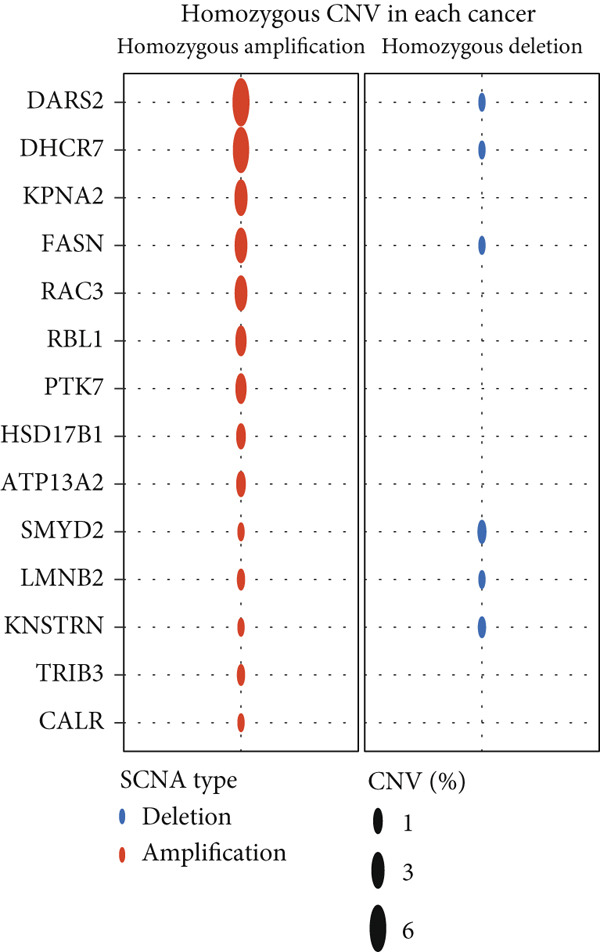
(d)

**Figure:**
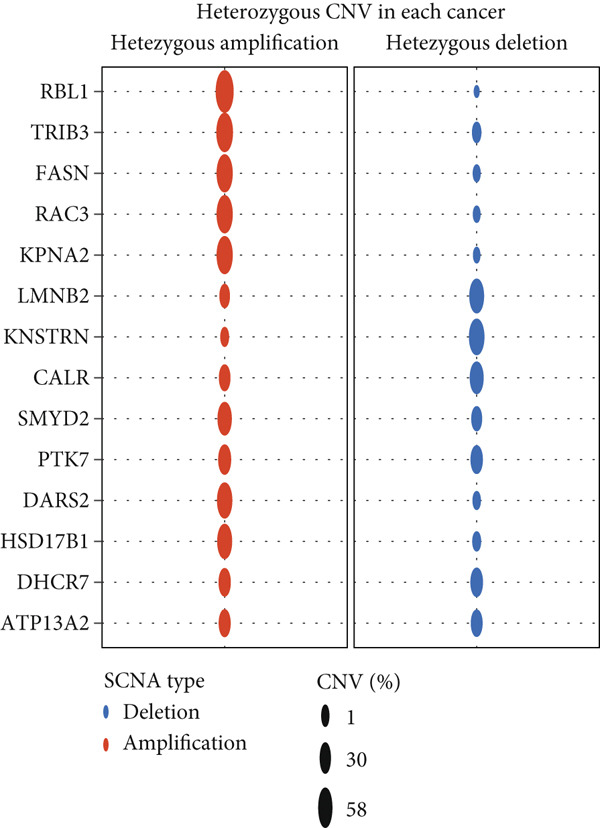
(e)

**Figure:**
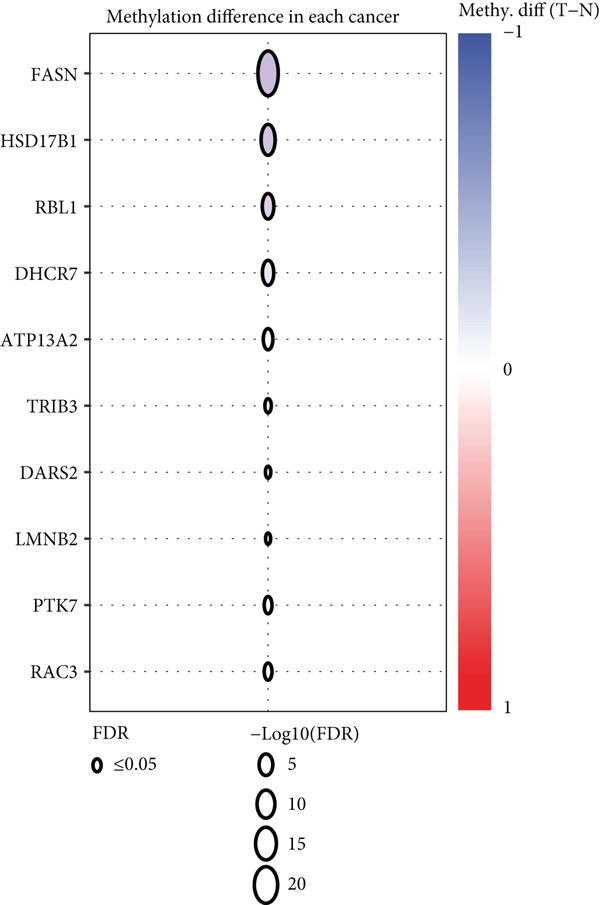
(f)

**Figure:**
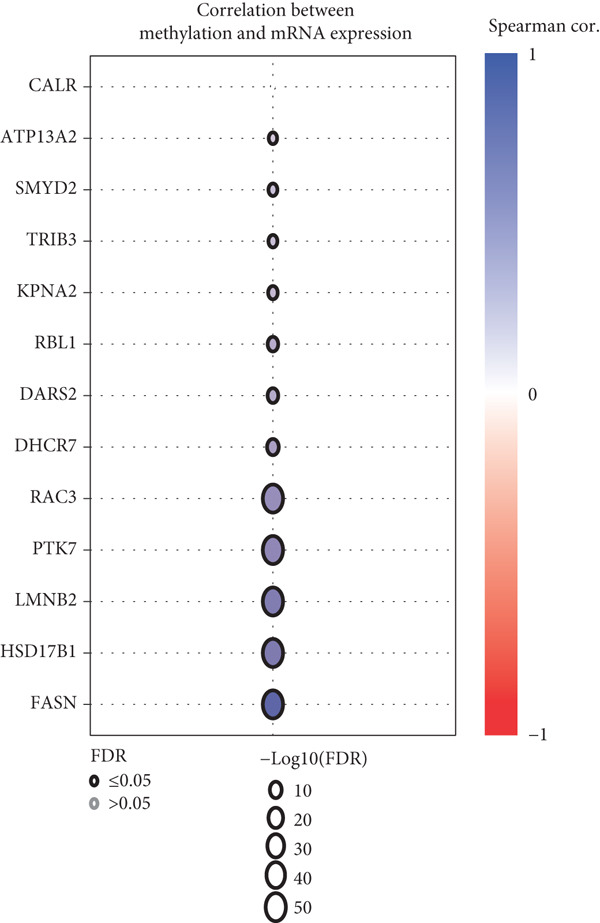
(g)

**Figure:**
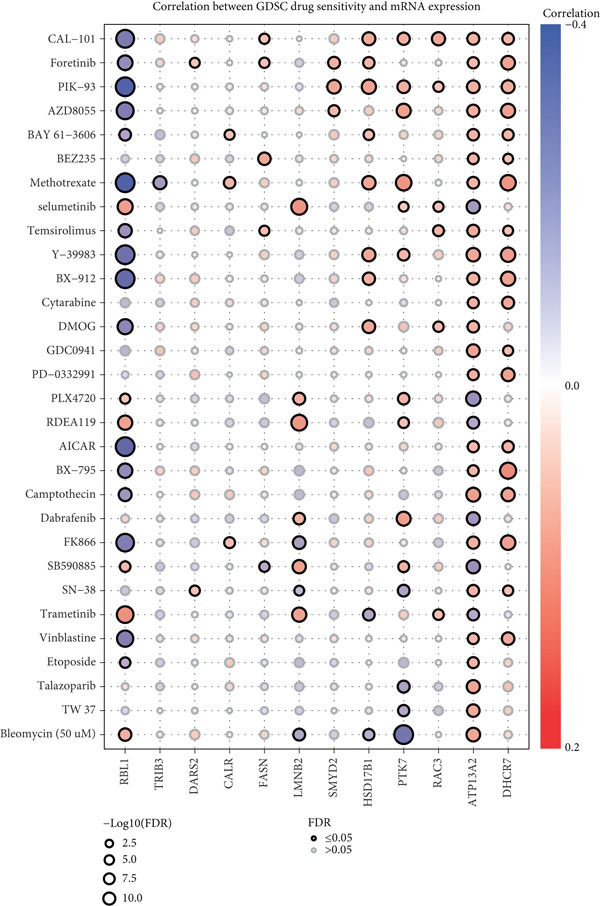
(h)

**Figure:**
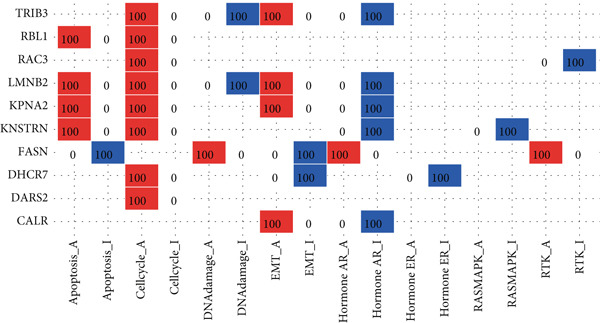
(i)

### 3.3. Molecular Typing

A clustering technique known as non‐NMF was employed to classify the samples from TCGA‐BLCA. To determine the optimal method for dividing TCGA‐BLCA samples into distinct subgroups for subsequent research, current evaluation metrics, which are based on cophenetic curves, are considered the most definitive approach. The optimal groupings are identified by observing the most significant drop point on the cophenetic curve. Our study revealed that dividing TCGA‐BLCA samples into two distinct groups, as indicated by the cophenetic curve, is the most appropriate strategy (Figure 3a,b). Subsequently, a prognostic analysis was conducted on patients from both Cluster 1 and Cluster 2, revealing that individuals in Cluster 1 consistently showed poorer prognoses, whether assessed by OS, PFS, or disease‐specific survival (DSS) (Figure 3c). The expression levels of particular genes related to stem cells in different clusters were analyzed, with results indicating significant differences and higher expression levels in Cluster 1 (Figure 3d).

Figure 3Patients with BLCA were categorized into two distinct clusters. (a) An assessment of the stability and effectiveness of the clusters was conducted using a range of methods. (b) A consensus plot demonstrating the results of NMF clustering. (c) Analysis of prognosis across the different clusters. (d) Evaluation of expression levels between the various clusters.  ^∗∗∗∗^ *p* < 0.0001.

**Figure:**
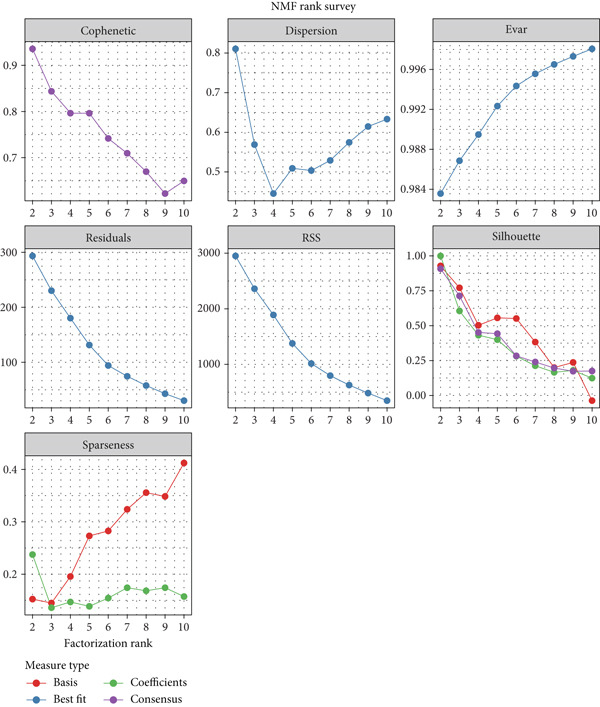
(a)

**Figure:**
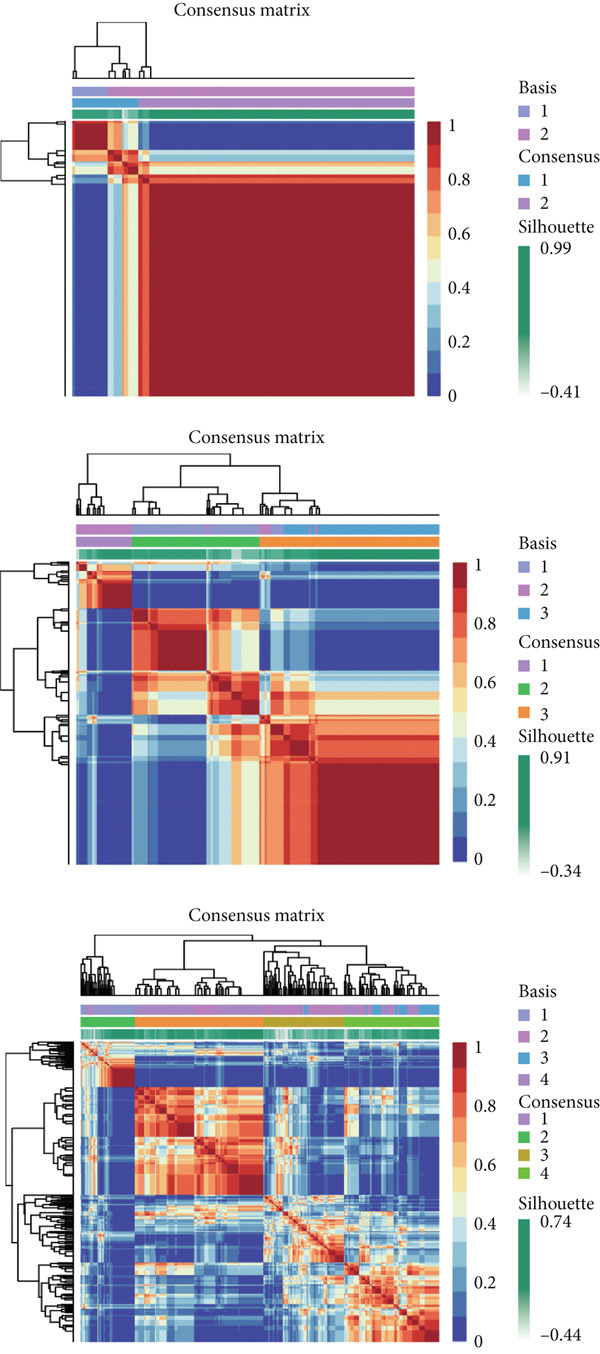
(b)

**Figure:**
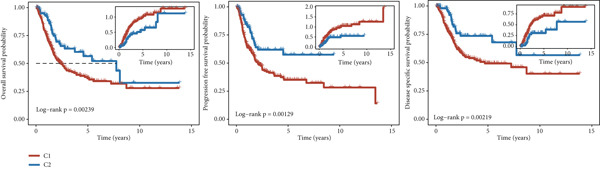
(c)

**Figure:**
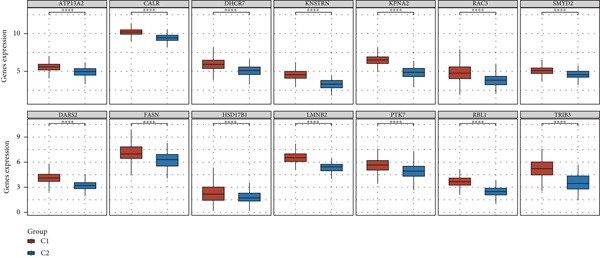
(d)

### 3.4. Correlation Analysis of Stem Cell–Related Genes With Immunotherapy and Chemotherapy in BLCA

Using the CIBERSORT algorithm, our study investigated the association between stem cell–related genes and immune cell infiltration in BLCA. In two distinct clusters, Cluster 2 exhibited higher scores for B‐cell memory, resting CD4+ memory T cells, regulatory T cells (Tregs), and activated mast cells. In contrast, elevated scores for activated CD4+ memory T cells, M0 macrophages, M1 macrophages, and resting mast cells were observed in Cluster 1 (Figure 4a,b). We also analyzed the expression differences of immune checkpoint–related genes between the two clusters, revealing significant discrepancies across all immune checkpoints evaluated in this research (Figure 4c). Furthermore, we depicted the levels of immune cell infiltration between the clusters using a heatmap generated by the CIBERSORT algorithm, alongside the proportion of tumor‐infiltrating immune cells within each sample (Figure 4d). An additional examination of the patients′ responses to immunosuppressive therapy indicated that Cluster 1 had a considerably higher tumor immune dysfunction and exclusion (TIDE) score compared to Cluster 2, which could explain the poorer prognosis observed in Cluster 1 (Figure 4e). Moreover, given that mitomycin is commonly used in the treatment of BLCA, we analyzed the differences in IC_50_ scores for mitomycin across the clusters. The results showed that Cluster 2 had a lower IC_50_ score for mitomycin (Figure 4f). Ultimately, we examined the factors contributing to the varied prognostic results observed in patients from Cluster 1 and Cluster 2 through KEGG analysis. Our findings suggested that Cluster 1 is predominantly linked to pathways, including the cell cycle, cellular senescence, and Human Immunodeficiency Virus 1 infection. Conversely, Cluster 2 is largely associated with steroid hormone biosynthesis, the PPAR signaling pathway, and the hedgehog signaling pathway (Figure 4g,h).

Figure 4Genes related to stem cells are linked to immunotherapy and chemotherapy in patients with BLCA. (a, b) Assessment of immune cell infiltration levels in BLCA samples using the CIBERSORT algorithm. (c) Comparison of immune checkpoint expression between two clusters. (d) Heatmap displaying variations in immune cell infiltration. (e) Evaluation of the response of the two clusters to immunosuppressive therapies. (f) Comparison of IC_50_ score differences for mitomycin across the two clusters. (g, h) KEGG pathway analysis.  ^∗∗∗∗^ *p* < 0.0001,  ^∗∗∗^ *p* < 0.001,  ^∗∗^ *p* < 0.01,  ^∗^ *p* < 0.05.

**Figure:**
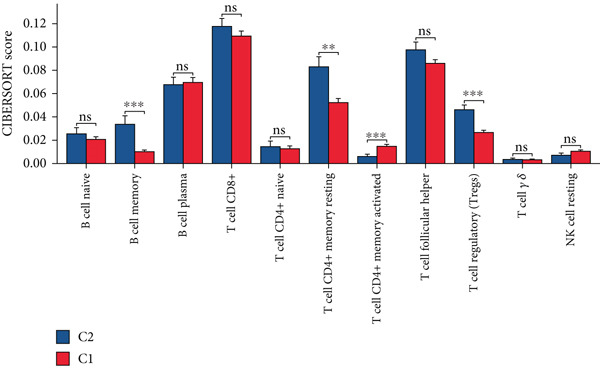
(a)

**Figure:**
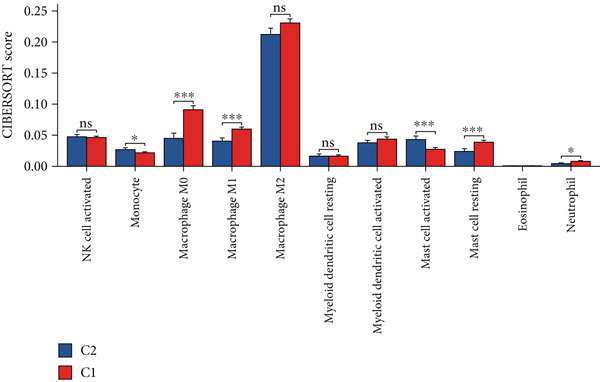
(b)

**Figure:**
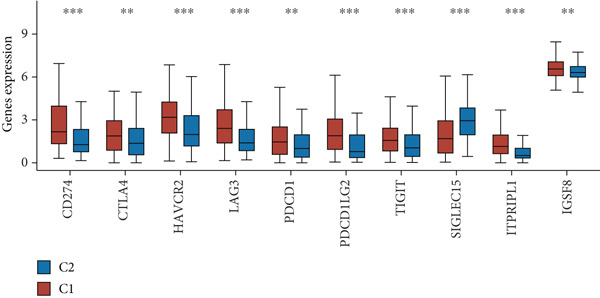
(c)

**Figure:**
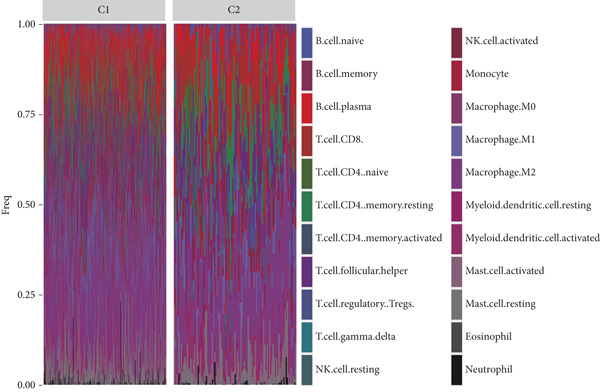
(d)

**Figure:**
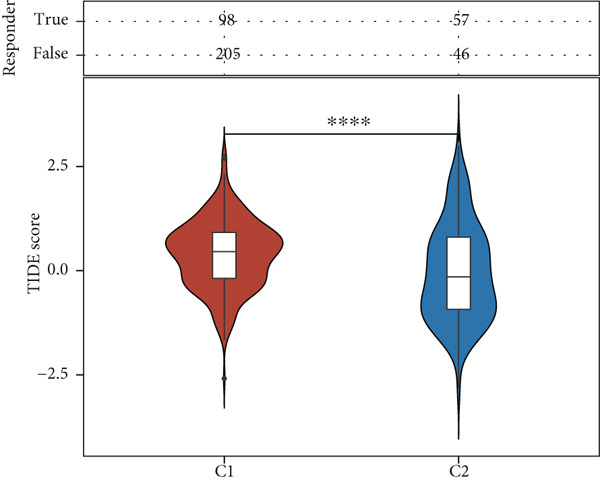
(e)

**Figure:**
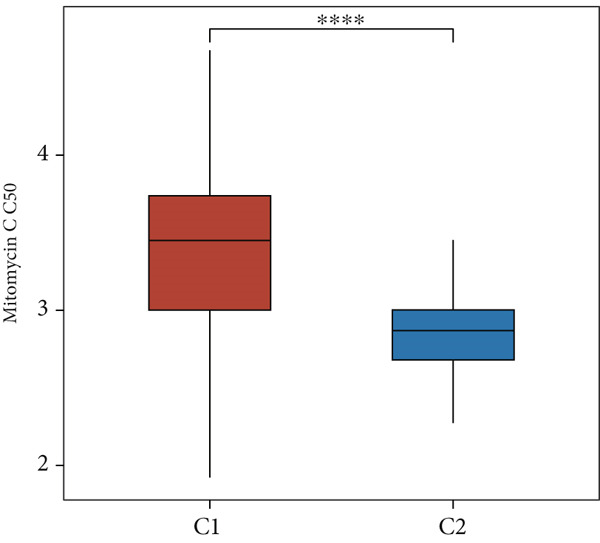
(f)

**Figure:**
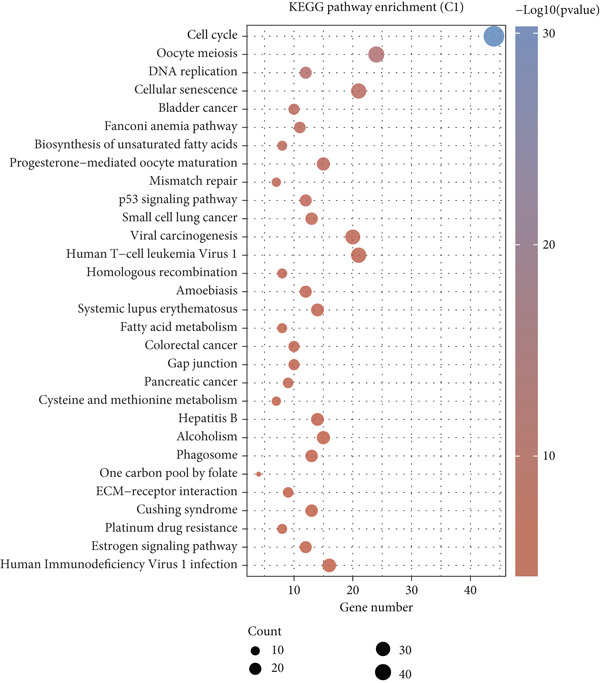
(g)

**Figure:**
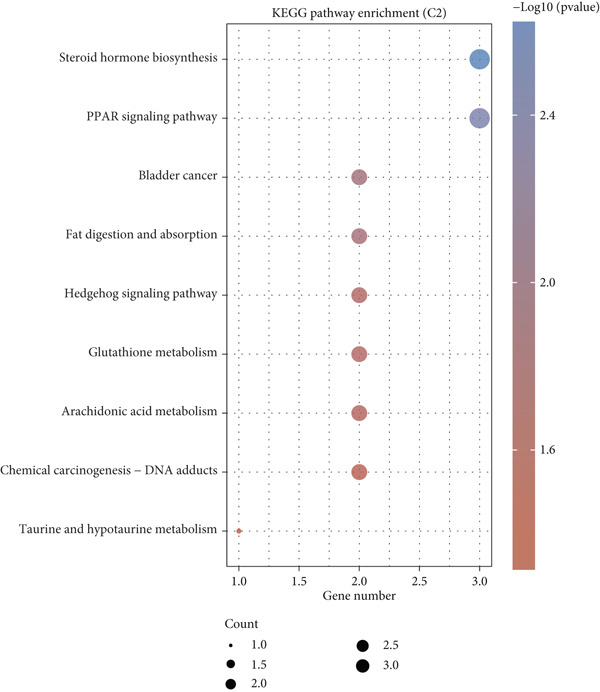
(h)

### 3.5. CALR Has Been Identified as a Key Stem Cell–Related Gene in BLCA

To further identify key genes among stem cell–related genes, we utilized prognostic data from the GSE13507 and TCGA‐BLCA datasets. Using the XGBoost algorithm, we ranked the correlation between stem cell–related genes and the prognosis of BLCA patients. The results indicated that CALR consistently ranked among the Top 5 genes (Figure 5a,b). Moreover, the GOSemSim algorithm, which prioritizes genes based on their similarity, identified CALR as the most significant gene (Figure 5c). Following this, we examined the relationship between CALR and immune infiltration in BLCA. Our findings revealed a positive association between CALR expression levels and activated memory CD4 T cells, M1 macrophages, M0 macrophages, and neutrophils. In contrast, CALR expression showed a negative relationship with activated dendritic cells, resting mast cells, plasma cells, and Tregs (Figure 5d,e). We also investigated the immune infiltration related to CALR using single‐cell databases (Figure 5f,g). Patients with low levels of CALR expression displayed better responses to treatment with immune checkpoint inhibitors (Figure 5h). The coexpression heatmap depicted the relationship between CALR and immune checkpoints (Figure 5i). Additionally, a molecular docking study was performed to assess the interaction between CALR and mitomycin, affirming that CALR exhibits a robust binding affinity for mitomycin (Figure 5j).

Figure 5The stem cell–related gene CALR is associated with immunotherapy for BLCA. (a, b) The XGBoost algorithm identifies key prognostic genes. (c) The GOSemSim algorithm ranks the importance of genes. (d–g) Correlation analysis between CALR and immune cell infiltration levels in BLCA. (h) Correlation analysis of CALR and immunotherapy responsiveness in BLCA. (i) Correlation analysis between CALR and immune checkpoints. (j) Analysis of the correlation between CALR and mitomycin.  ^∗∗∗∗^ *p* < 0.0001.

**Figure:**
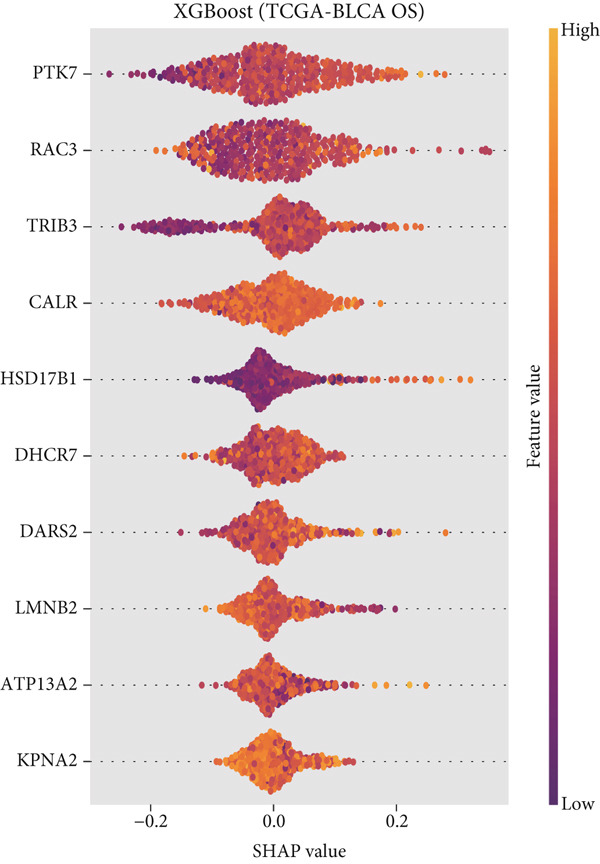
(a)

**Figure:**
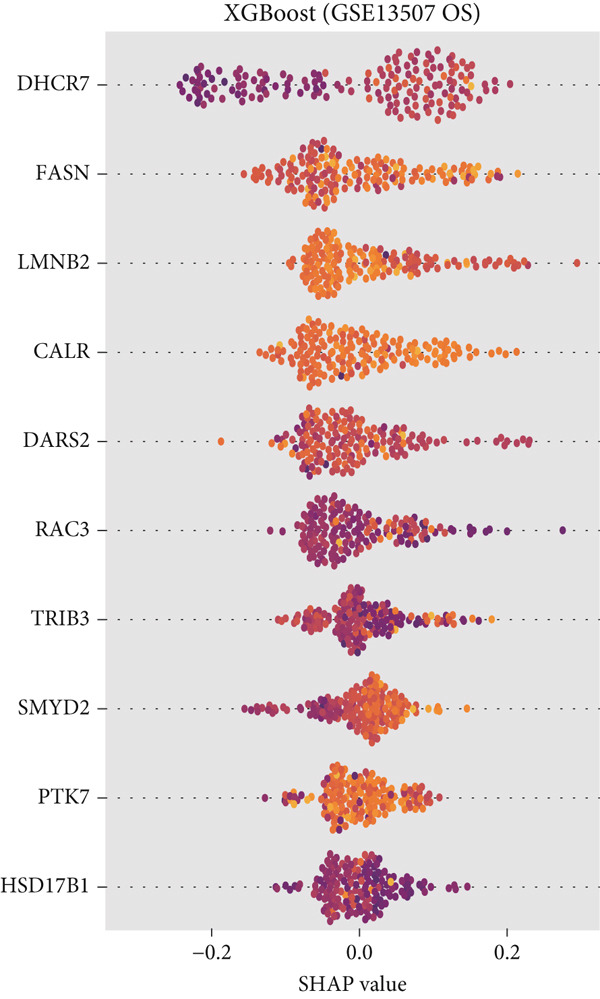
(b)

**Figure:**
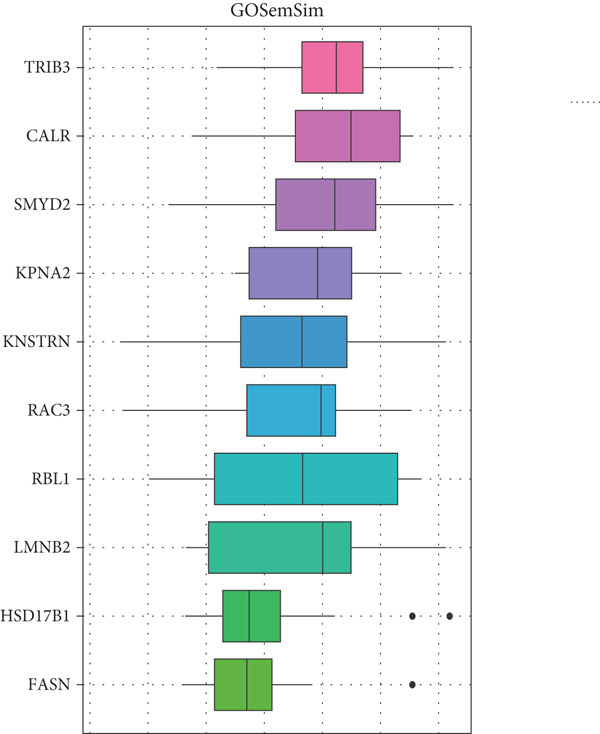
(c)

**Figure:**
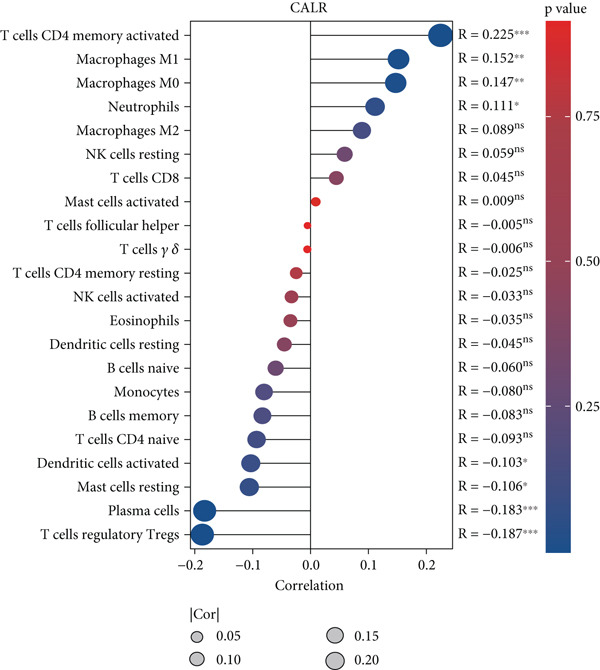
(d)

**Figure:**
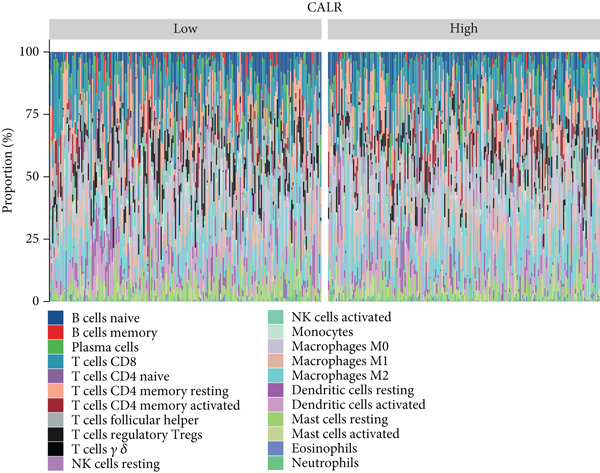
(e)

**Figure:**
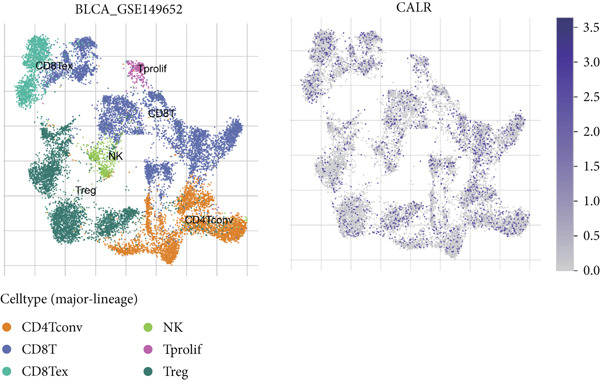
(f)

**Figure:**
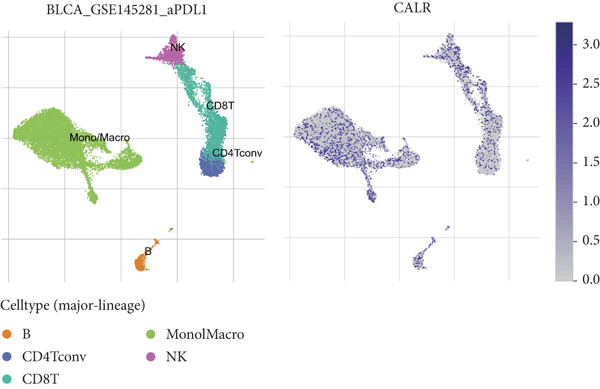
(g)

**Figure:**
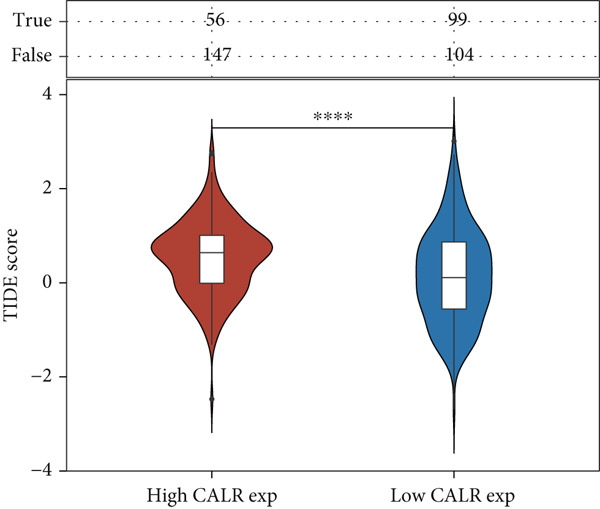
(h)

**Figure:**
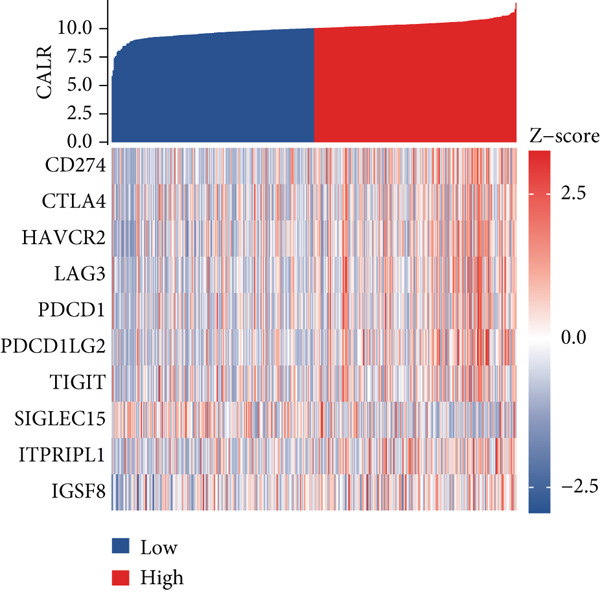
(i)

**Figure:**
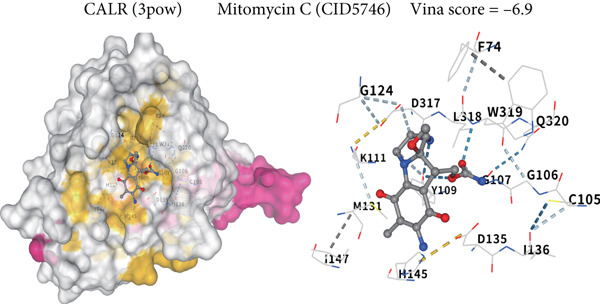
(j)

### 3.6. CALR Is Highly Expressed in BLCA

The differences in CALR expression between paired and unpaired samples from the TCGA‐BLCA dataset were analyzed, revealing a significant upregulation of CALR in BLCA (Figure 6a,b). Furthermore, an increase in CALR expression was observed in high‐grade BLCA, showing a marked elevation in patients who had passed away compared to survivors (Figure 6c,d). We also evaluated the potential of CALR expression as a diagnostic marker in BLCA patients, with the ROC curve demonstrating considerable diagnostic promise (Figure 6e). In addition, we investigated the prognostic relevance of CALR in BLCA patients, finding that those with higher levels of CALR expression had worse prognoses (Figures 6f, 6g, and 6h). We also analyzed CALR expression levels in various BLCA cell lines utilizing the CCLE database (Figure 6i). Data from the Human Protein Atlas database revealed that CALR protein levels were significantly elevated in BLCA tissues compared to normal bladder tissues, primarily localized at the cell membrane (Figure 6j,k).

Figure 6Patients with high CALR expression have a poor prognosis. (a, b) The variation in CALR expression between cancerous and adjacent tissue samples. (c) The differences in CALR expression observed between high‐grade and low‐grade BLCA. (d) The contrast in CALR expression among BLCA survivors compared to nonsurvivors. (e) The diagnostic predictive value of CALR in BLCA. (f–h) The prognostic importance of CALR in BLCA. (i) Variations in CALR expression in BLCA cell lines. (j) The expression levels of CALR protein in BLCA specimens show differences. (k) Analysis of CALR protein localization within tumor cells.  ^∗∗∗^ *p* < 0.001,  ^∗∗^ *p* < 0.01.

**Figure:**
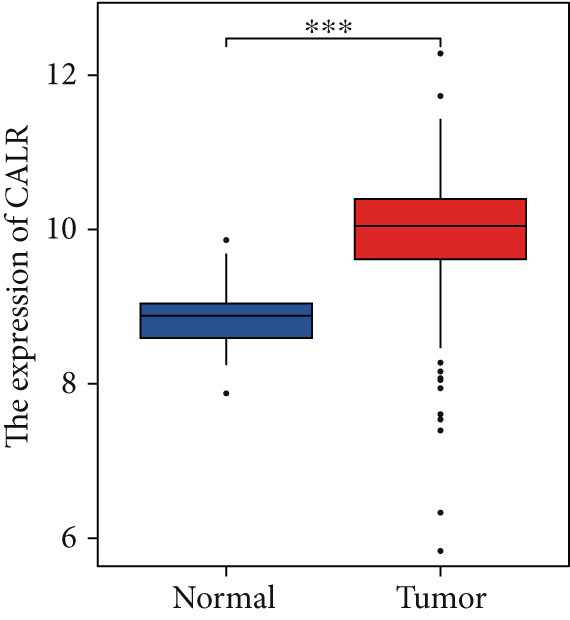
(a)

**Figure:**
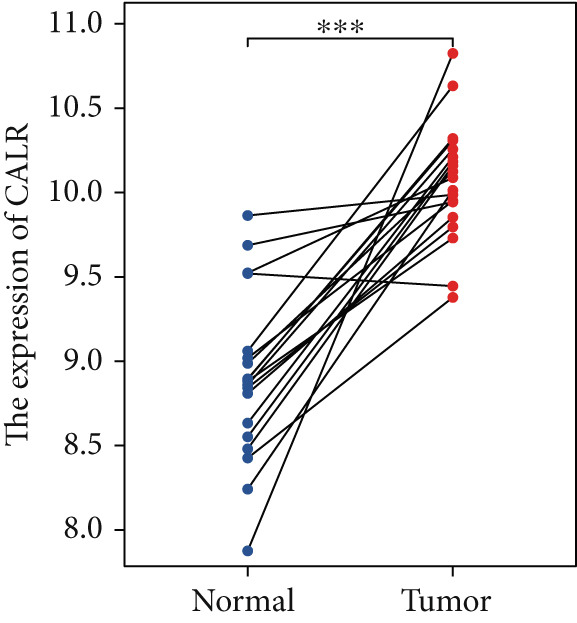
(b)

**Figure:**
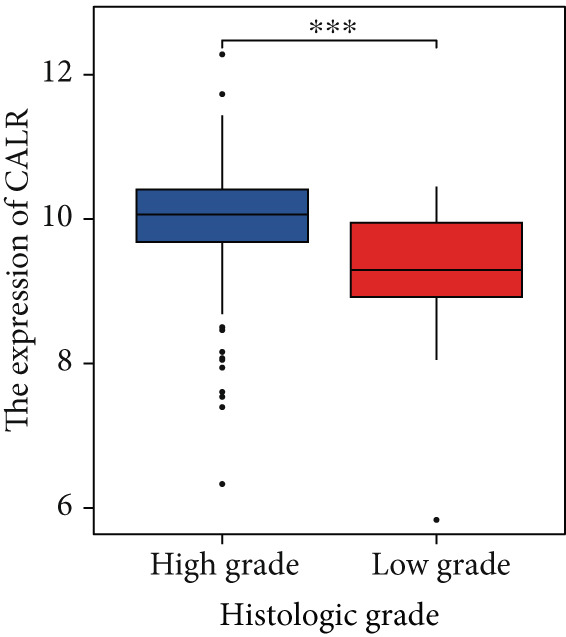
(c)

**Figure:**
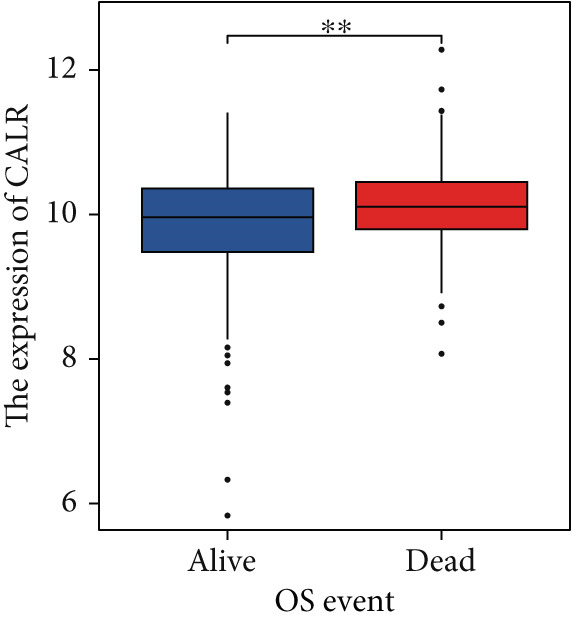
(d)

**Figure:**
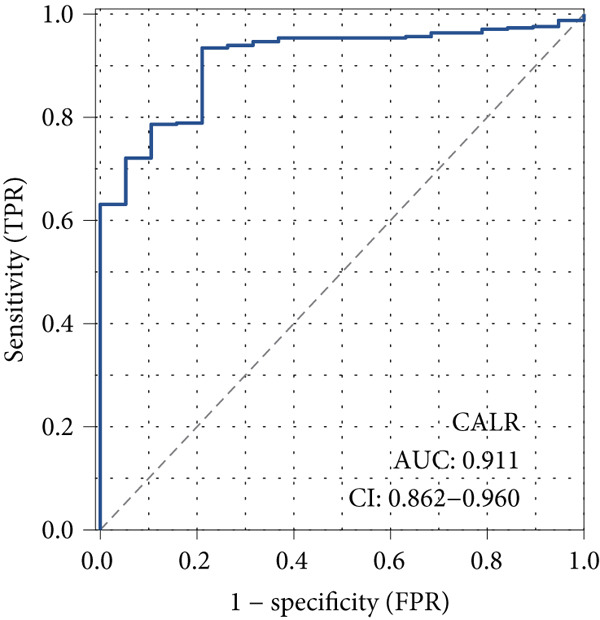
(e)

**Figure:**
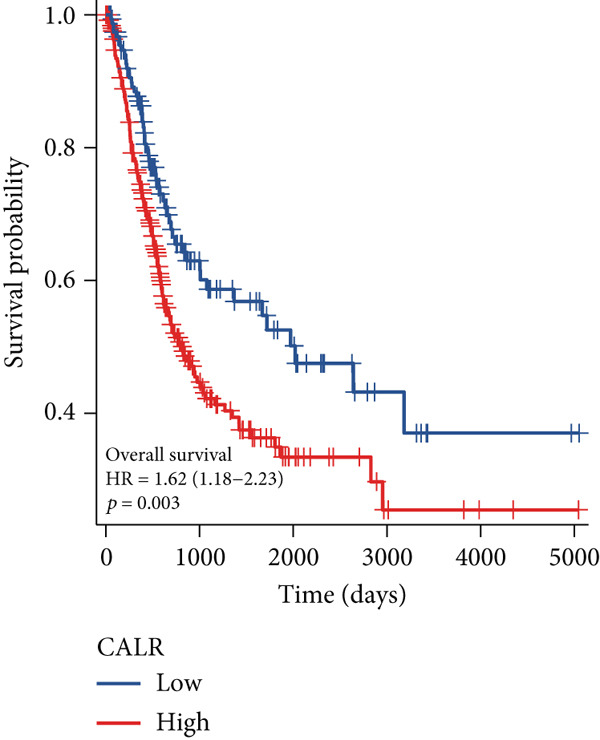
(f)

**Figure:**
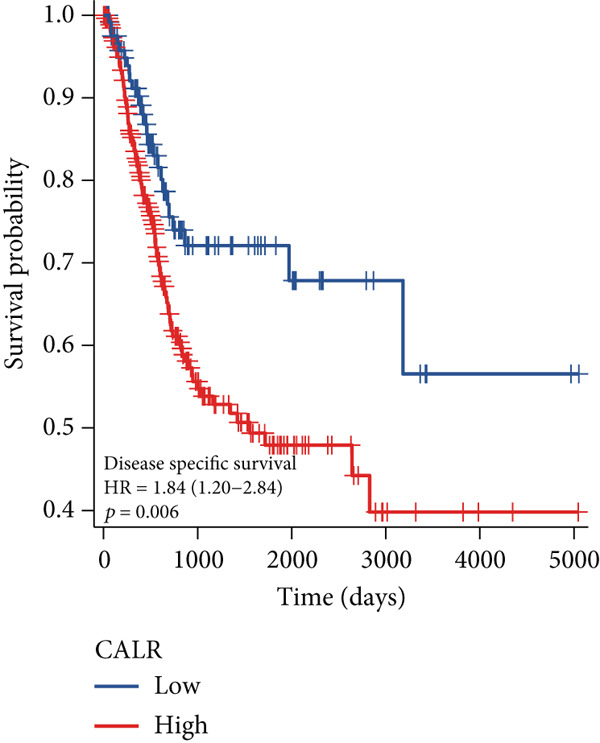
(g)

**Figure:**
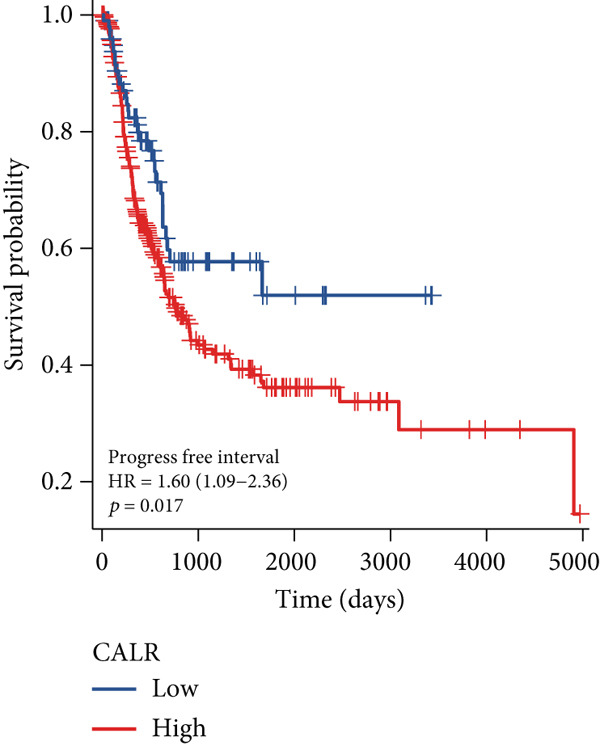
(h)

**Figure:**
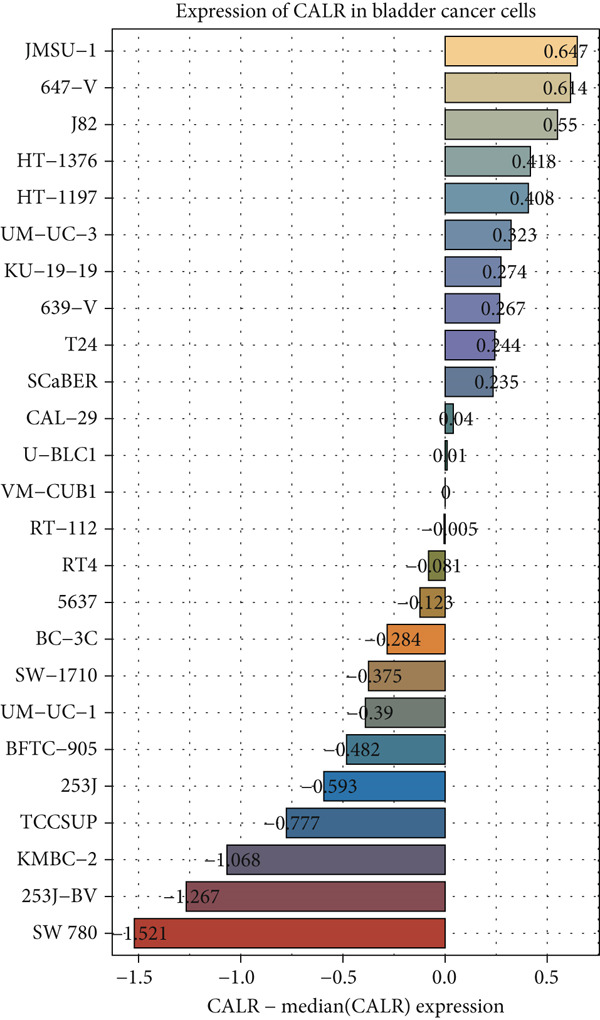
(i)

**Figure:**
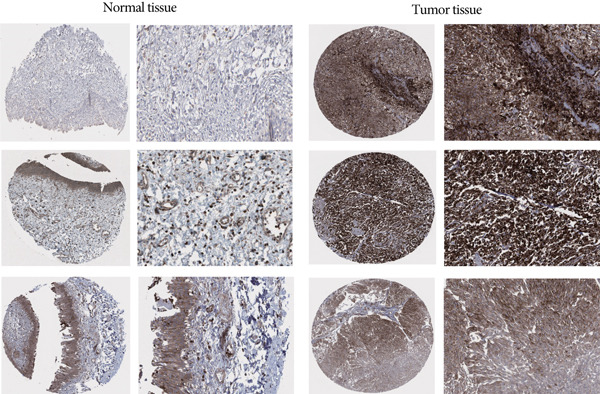
(j)

**Figure:**
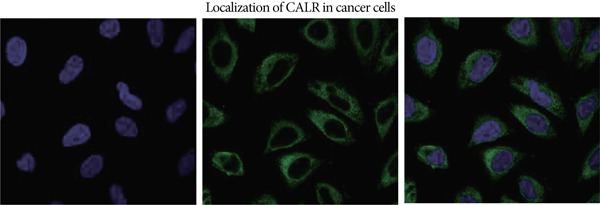
(k)

### 3.7. Downregulated CALR Repressed TCC Cell Migration and Invasion and Inhibited Cell Proliferation In Vitro

To clarify the function of CALR in the advancement of BLCA cells, we transiently diminished CALR expression in BLCA cell lines using siRNA interference techniques, establishing interference cell lines (647‐V CALR‐si and J82 CALR‐si) and evaluating CALR expression levels. The transfection efficiency was assessed using RT‐qPCR. Results showed that CALR expression in the CALR‐si1 group decreased approximately fourfold compared to pretransfection levels, indicating significant knockdown in both 647‐V and J82 cells (Figure 7a; *p* = 0.005, *p* < 0.0001). No significant differences were observed in the other control groups. We examined how CALR gene expression influences cell migration and invasion using both the wound healing assay and Transwell assays. For the wound healing assay, cells were monitored and photographed every 24 h based on experimental design and growth patterns. By capturing images for analysis and comparison, we observed that after 24 h of culture, the migration capabilities of cells in the CALR‐suppressed group (647‐V CALR‐si and J82 CALR‐si) were significantly decreased relative to the blank and negative control groups (*p* = 0.003, Figure 7b; *p* = 0.028, Figure 7c). Additionally, Transwell assays revealed that the number of cells in the CALR knockdown group (647‐V CALR‐si and J82 CALR‐si) that migrated through the chamber, with or without Matrigel, was notably less than that in the negative control (*p* < 0.05, Figure 7d,e). As illustrated in Figure 7j, the sphere formation assay indicated that the downregulation of CALR influences the proliferation capacity, self‐renewal efficiency, and long‐term survival of BLCA stem cells. The findings revealed that, when compared to the NC group, there was a noticeable reduction in the size of stem cell spheres and a significant decrease in structural density in the group with CALR downregulation (*p* = 0.007, *p* = 0.002; Figure 7k). Based on these findings, we performed immunofluorescence assays to assess the expression levels of SOX2 and POU5F1, markers of stem cell proliferation. Quantitative analysis revealed significantly decreased expression of both factors following CALR knockdown. Indicative of stemness loss, SOX2 mean fluorescence intensity decreased from 128.4 ± 12.3 (NC‐si) to 68.9 ± 9.1 (CALR‐si) (*p* = 0.0014, *p* = 0.0009; Figure 7f,g; *p* = 0.0023, *p* = 0.0006; Figure 7h,i). Our results indicate that CALR is involved in maintaining the stem cell–like characteristics of BLCA cells and plays a critical role in the progression of BLCA.

Figure 7Knockdown of CALR inhibits the proliferation and invasion of BLCA cells in vitro. (a) qPCR was performed to assess the transfection efficiency of siRNA in both 647‐V and J82 cell lines. (b, c) Wound healing assays were conducted to evaluate the migration potential of 647‐V and J82 cells across various conditions. (d) A Transwell assay was utilized to confirm how the downregulation of CALR affects the invasion and migration capabilities of J82 cells in vitro. (e) Another Transwell assay assessed the impact of CALR suppression on the invasion and migration abilities of 647‐V cells in vitro. (f, g) Immunofluorescence studies demonstrated the influence of CALR downregulation on SOX2 expression in the nuclei of BLCA cells. (h, i) Another immunofluorescence studies demonstrated the influence of CALR downregulation on POU5F1 expression in the nuclei of BLCA cells. (j, k) The ability of 647‐V and J82 cells to form spheroids was evaluated after CALR downregulation.  ^∗∗∗∗^ *p* < 0.0001,  ^∗∗∗^ *p* < 0.001,  ^∗∗^ *p* < 0.01.

**Figure:**
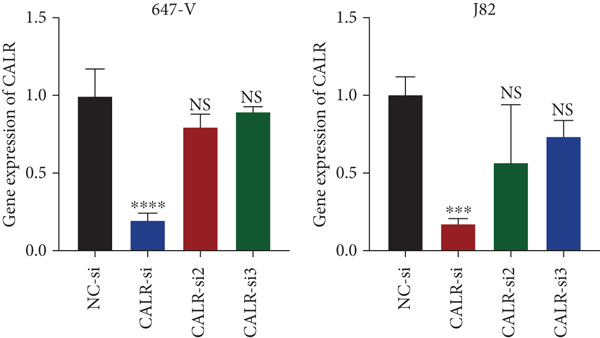
(a)

**Figure:**
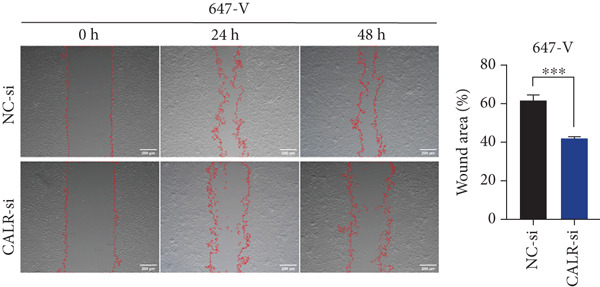
(b)

**Figure:**
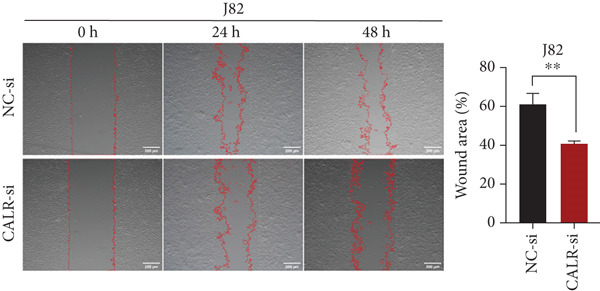
(c)

**Figure:**
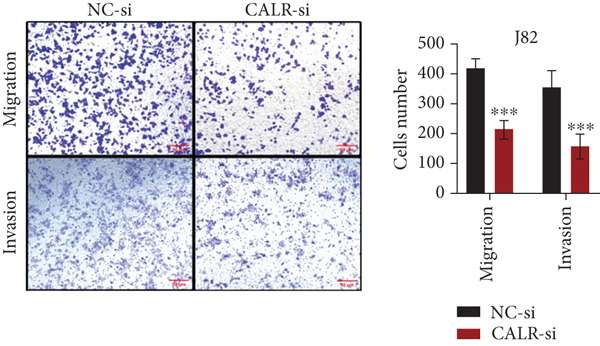
(d)

**Figure:**
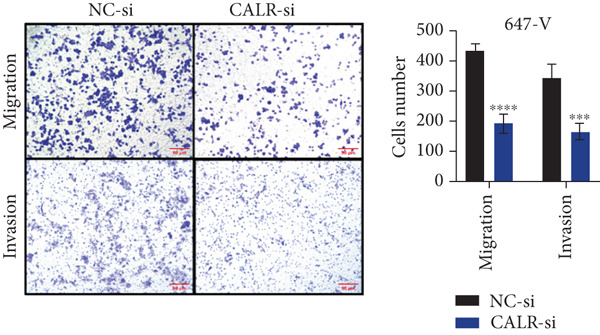
(e)

**Figure:**
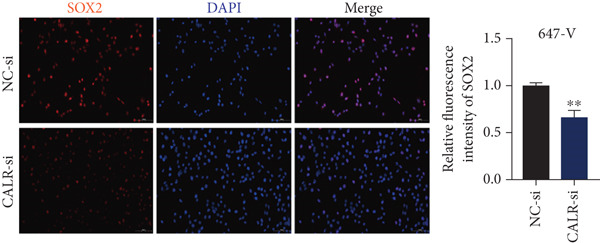
(f)

**Figure:**
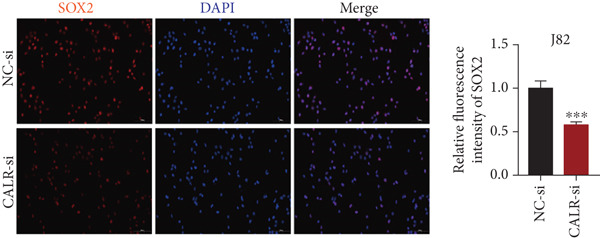
(g)

**Figure:**
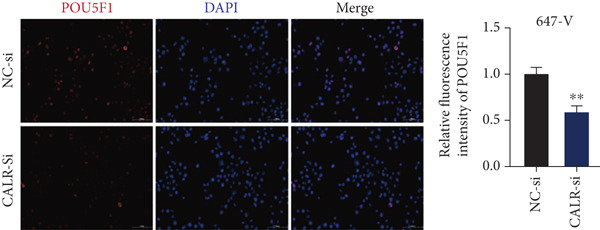
(h)

**Figure:**
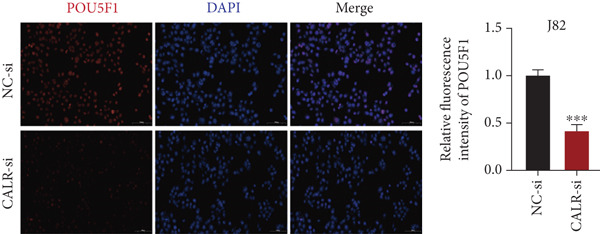
(i)

**Figure:**
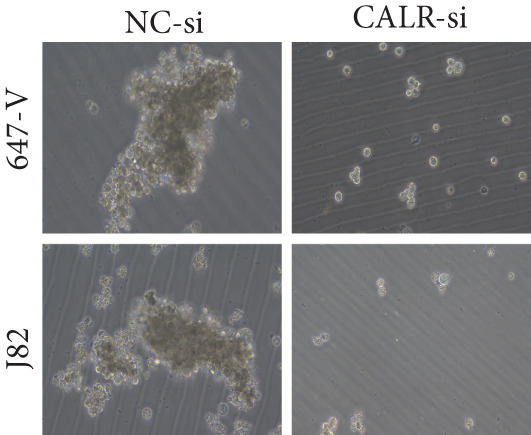
(j)

**Figure:**
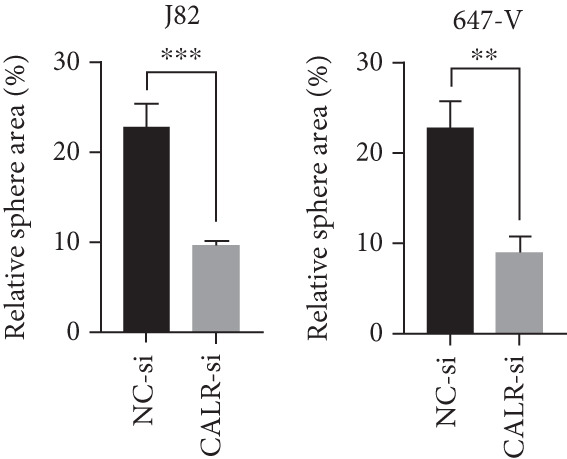
(k)

## 4. Discussion

BLCA, a prevalent malignant tumor of the urinary system, has demonstrated an increasing trend in both incidence and mortality rates globally. Projections indicate that by 2040, the number of new cases and deaths will rise to 991,000 and 397,000, respectively [18]. This upward trend is partly attributed to the prevalence of risk factors such as smoking [19]. To address the high incidence and mortality rates of BLCA, researchers are actively investigating new biomarkers aimed at enhancing early diagnosis and treatment outcomes. Currently, although various urinary biomarkers are utilized for the detection and monitoring of BLCA, their sensitivity and specificity remain suboptimal [20]. For instance, FDA‐approved urine tests such as BTA stat and NMP22 exhibit significant variability in sensitivity and specificity across different studies, which restricts their application in clinical practice [21]. Nevertheless, new biomarkers continue to face numerous challenges in clinical application, including the lack of multicenter clinical validation and standardized testing methods [22]. Therefore, future research should concentrate on improving the accuracy and clinical applicability of biomarker detection to better facilitate early detection and treatment monitoring of BLCA [23]. In conclusion, the increasing rates of BLCA incidence and mortality pose a major challenge to public health worldwide.

CSCs are essential in the progression, development, and treatment resistance of BLCA. Studies show that these cells can self‐renew and differentiate into different cell types, making them important factors in tumor metastasis and recurrence [4]. Furthermore, the presence of CSCs is considered one of the primary factors leading to drug resistance in BLCA, particularly against conventional treatments such as chemotherapy and radiotherapy [24]. In the immunotherapy of BLCA, targeting CSCs represents a promising strategy. By identifying and targeting specific antigens associated with CSCs, the effectiveness of immunotherapy can be substantially improved. For instance, natural killer (NK) cells have been shown to effectively recognize and eliminate CSCs, providing new insights into the immunotherapeutic approaches for BLCA [25]. Moreover, research has shown that improving the immune response by altering immune cells in the tumor microenvironment, including macrophages and dendritic cells, can further boost the effectiveness of immunotherapy [26–28]. In recent years, immunotherapy strategies targeting CSCs have made significant advancements. Techniques such as adaptive T‐cell therapy, dendritic cell vaccines, oncolytic viruses, and immune checkpoint inhibitors have been employed to target CSCs, yielding promising outcomes [29]. Additionally, chemokines within the tumor microenvironment are recognized as key elements that affect the success of immunotherapy. By fine‐tuning these factors, the overall impact of immunotherapy can be greatly improved [30]. In summary, CSCs are crucial in immune evasion and resistance to treatment in BLCA. A comprehensive exploration of the interaction between CSCs and the immune system may lead to innovative strategies and techniques for the immunotherapy of BLCA.

In our research, we first pinpointed genes linked to BLCA stem cells. By employing the GSCA platform [31], we investigated the possible functions of these 14 genes in BLCA. Our analysis demonstrated that stem cell–related genes are predominantly linked to the cell cycle and EMT in BLCA. In the TCGA‐BLCA dataset, a total of 14 stem cell–related genes were used to group the BLCA samples by applying the NMF algorithm [32]. The cophenetic curve suggested that the best categorization occurred when the BLCA samples were split into two distinct clusters. Importantly, patients within Cluster 1 regularly showed the least favorable prognosis. To investigate the factors contributing to the differences in prognosis among these individuals, the TIDE algorithm was employed to assess the response to immune checkpoint inhibitor therapy across the clusters. The results indicated that individuals in Cluster 1 demonstrated higher TIDE scores and less positive responses to immunotherapy, potentially accounting for the unfavorable prognosis observed in this group. Utilizing various machine learning techniques, we determined CALR to be a crucial stem cell–associated gene in BLCA and validated the relationship between CALR and BLCA stem cells through in vitro studies. While somatic mutations in CALR are most famously established as key drivers in certain myeloproliferative neoplasms (MPNs), its role in solid tumors like bladder cancer is less direct and still under investigation [33]. Research suggests that CALR expression may be dysregulated in bladder cancer tissues. Overexpression of calreticulin has been observed in some bladder cancer studies and correlates with features like advanced stage and potentially poorer prognosis [34, 35]. Proposed mechanisms include promoting cancer cell proliferation, migration, and invasion. Importantly, calreticulin′s role as an “eat‐me” signal during immunogenic cell death (ICD) is a key area of interest. Its exposure on the surface of dying cancer cells can enhance antitumor immune responses, potentially influencing the efficacy of therapies like chemotherapy or radiotherapy in bladder cancer. Further research is needed to fully elucidate CALR′s specific contributions to bladder cancer and its potential as a biomarker or therapeutic target.

## 5. Conclusion

Our research revealed an essential regulatory link between CALR and stem cells associated with BLCA, utilizing several machine learning approaches alongside in vitro experiments. Additionally, CALR may act as a prognostic biomarker for BLCA.

## Ethics Statement

The authors have nothing to report.

## Consent

The authors have nothing to report

## Disclosure

All authors have read and approved the final manuscript.

## Conflicts of Interest

The authors declare no conflicts of interest.

## Author Contributions

Zhihao Ling drafted the manuscript; Shuo He, Tianyu Li, and Jiandong Zhang conducted preliminary investigations. Beibei Liu was responsible for reviewing it.

## Funding

No funding was received for this manuscript.

## Data Availability

The data that support the findings of this study are available from the corresponding author upon reasonable request.
